# Lacunar Infarction as a Neurological Manifestation of Human Immunodeficiency Virus (HIV): A Case Report

**DOI:** 10.7759/cureus.111735

**Published:** 2026-06-29

**Authors:** Ziryab Imad Taha Mahmoud, Alfadil Mohammed, Yassin A Abdalla, Omer Ibrahim Ali Ibrahim, Mohammed Osman Ahmed Osman

**Affiliations:** 1 Internal Medicine, University of Bahri, Khartoum, SDN; 2 Internal Medicine, National Ambulance, Abu Dhabi, ARE; 3 Internal Medicine, Omdurman Islamic University, Khartoum, SDN; 4 Radiology, University of Khartoum, Khartoum, SDN; 5 Internal Medicine, National Ribat University, Khartoum, SDN

**Keywords:** acute ischaemic stroke, case report, hiv aids, lacunar infarction, small vessel disease

## Abstract

Human immunodeficiency virus (HIV) belongs to the *Lentivirus *genus of the *Retroviridae *family. The virus primarily attacks CD4+ T-lymphocyte helper cells, which results in severe immunological suppression and ongoing cell death. HIV has several neurological manifestations, including meningitis, epileptic seizures, and stroke.

A 35-year-old male presented with an eight-month history of fever, convulsion, and weight loss. Neurological exam was significant for bilateral upgoing plantar response and neck stiffness. The enzyme-linked immunosorbent assay (ELISA) was positive for HIV, and his brain MRI revealed a hyperintense signal in the head of the right caudate nucleus, right basal ganglia, left basal ganglia, and left frontoparietal subcortical white matter, suggestive of an acute lacunar infarct.

HIV patients with neurological complaints, especially those with convulsions, require careful evaluation and direct input from neurologists and infectious disease specialists. HIV as a potential cause of stroke in the young population, especially in low-income countries, should not be overlooked due to its notorious effect on the central nervous system (CNS) and overall health.

## Introduction

Human immunodeficiency virus (HIV) is a positive-sense single-stranded RNA virus that belongs to the family of retroviridae. It has two subtypes: HIV-1, which is the most prevalent, and HIV-2, which is mainly found in West Africa. HIV primarily targets CD4+ T-lymphocytes, causing profound immunological suppression, which eventually leads to acquired immune deficiency syndrome (AIDS) if left untreated. It is estimated that there are 40.8 million HIV-infected people in 2024, of which two-thirds live in Africa [1,2].

The development of neurological conditions in people infected with HIV is influenced by host genetic makeup, environmental factors, and HIV properties. These conditions can be classified into three broad categories: primary HIV neurological diseases, secondary or opportunistic neurological diseases, and treatment-related neurological diseases, such as immune reconstitution inflammatory syndrome. From these, meningoencephalitis or acute aseptic (viral) meningitis is the most frequent neurologic condition linked to the initial HIV infection [3].

Neurological manifestations of HIV include hemiplegia, headache, cranial nerve impairment, epileptic seizures, memory disturbance, coma, ataxia, confusion, and tremor [4].

Stroke is defined as an acute focal neurological deficit due to vascular origin. It is divided into ischemic and hemorrhagic strokes. Ischaemic stroke is further divided into five main categories. HIV-related strokes have become a prominent sequelae of HIV with unfavorable outcomes in recent years. [5]. Young persons (those under 45) with HIV infection had a greater incidence of all types of stroke, which emphasizes the need to rapidly and aggressively test for and manage risk factors [6].

Different mechanisms were hypothesized to cause stroke in HIV patients, including coagulopathy, vasculopathy, infections, and thrombosis [5]. In a comparative study done in patients diagnosed with stroke in Malawi, HIV-positive individuals were considerably younger with less prominent vascular risk factors. HIV-associated vasculopathy was the most frequent cause, followed by opportunistic infections. It is worth mentioning that stroke occurred in 25% of patients shortly after beginning antiretroviral therapy (ART), which may indicate an immunological reconstitution-like syndrome [7].

Our case shows a rare and clinically significant presentation of HIV in a relatively young patient with no vascular risk factors. It also highlights the possibility that multifocal lacunar infarcts may be the only and early manifestation of HIV infection. The presentation of convulsions makes the case interesting, as they are not a common presentation in patients with cerebral small-vessel disease.

Written informed consent was obtained from the patient to publish this case report, and the patient was informed that their identity will be kept anonymous.

## Case presentation

A 35-year-old previously healthy male presented in March 2024 with an eight-month history of fever, generalized tonic-clonic seizures, and significant weight loss. He was a non-smoker with no history of diabetes mellitus or hypertension.

On examination, he was vitally stable and was neither pale nor jaundiced. His Glasgow Coma Scale (GCS) score was 15/15. Neurological examination revealed bilateral extensor plantar responses. Muscle power was graded as 4+/5 in both upper and lower limbs. Muscle tone, sensation, and coordination were normal. Deep tendon reflexes were preserved, and cranial nerve examination was unremarkable. Skin rashes were noted over the lower extremities.

Laboratory investigations demonstrated normocytic normochromic anemia, leukopenia, and an elevated erythrocyte sedimentation rate (ESR). Serum albumin was mildly reduced, while liver function tests and coagulation profile were within normal limits. The patient had normal sodium, potassium, urea, and creatinine levels. Viral screening was positive for HIV, and enzyme-linked immunosorbent assay (ELISA) testing was strongly reactive (Table 1).

**Table 1:** Blood workup APTT - activated partial thromboplastin time; BFFM - blood film for malaria; CRP - C-reactive protein; ESR - erythrocyte sedimentation rate; INR - International Normalized Ratio; PT - prothrombin time; WBC - white blood cell

Variables	Result	Reference Range (Adult Male)
Hemoglobin	11.8 g/dL	12.0 - 16.0 g/dL
Platelets	156 ×10³/µL	150 - 400 ×10³/µL
Total WBC	2.7 ×10³/µL (PMN 79%, Lymph 11%)	4 - 11 ×10³/µL
Serum creatinine	0.7 mg/dL	0.6 - 1.1 mg/dL
Blood urea	26 mg/dL	15 - 50 mg/dL
Serum potassium	3.0 mmol/L	3.6 - 5.5 mmol/L
ESR	60 mm/hr	Up to 10 mm/hr
CRP	9.7 mg/L	Negative (<10 mg/L)
Total protein	6.9 g/dL	6.4 - 8.3 g/dL
Albumin	3.1 g/dL	3.5 - 5.0 g/dL
Globulin	3.6 g/dL	2.0 - 3.5 g/dL
BFFM	Negative	Negative
APTT	23.6 sec	25- 35 seconds
PT	18.7 sec	11- 15 seconds
INR	1.2	0.8 - 1.2

An initial computed tomography (CT) scan of the brain was unremarkable, excluding large space-occupying lesions (Figure 1). However, magnetic resonance imaging (MRI) of the brain performed one week later revealed bilateral hyperintense signals involving the basal ganglia and the left frontoparietal subcortical white matter, suggestive of acute multifocal lacunar infarctions (Figures 2-5). No significant post-contrast enhancement was observed on T1-weighted images.

**Figure 1:**
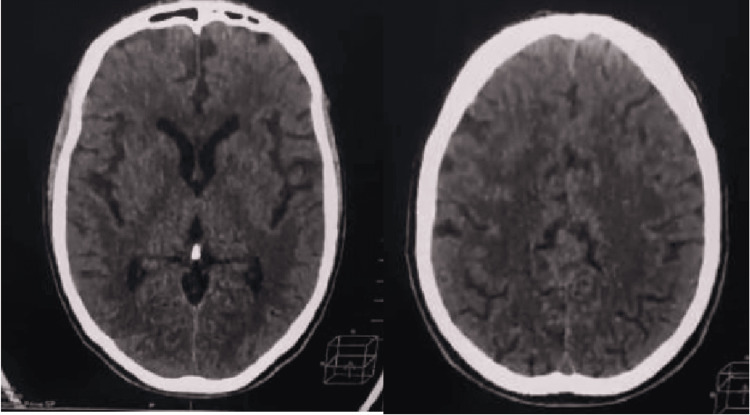
Axial non-contrast enhanced CT brain at the level of the basal ganglia and centrum semiovale shows no significant abnormality

**Figure 2:**
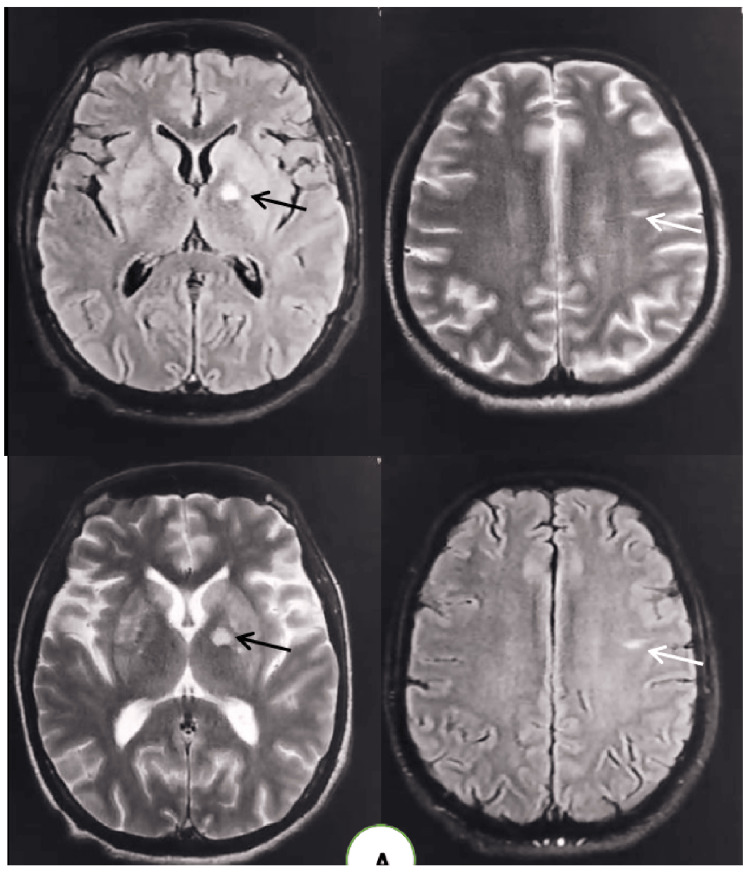
Selected axial cuts of T2-weighted imaging and FLAIR at basal ganglia level (left) and centrum semiovale level (right) demonstrate bilateral basal ganglia (black arrow) and left frontal subcortical white matter (white arrow) foci of high signal intensity FLAIR - fluid-attenuated inversion recovery

**Figure 3:**
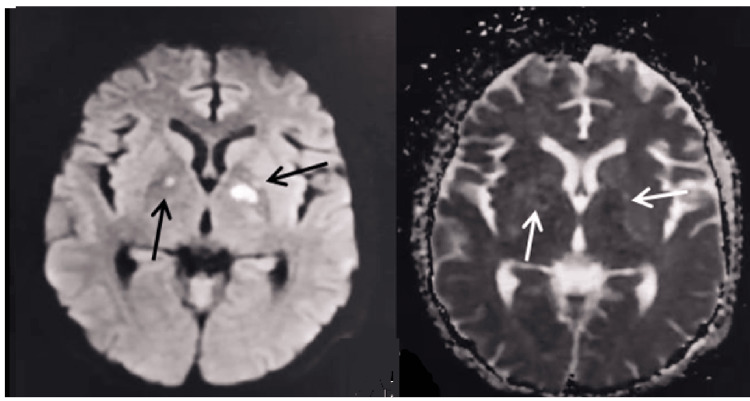
Subcortical and basal ganglia foci show diffusion restriction, indicated by black arrows on diffusion-weighted imaging and white arrows on the apparent diffusion coefficient map, suggestive of acute lacunar infarcts

**Figure 4:**
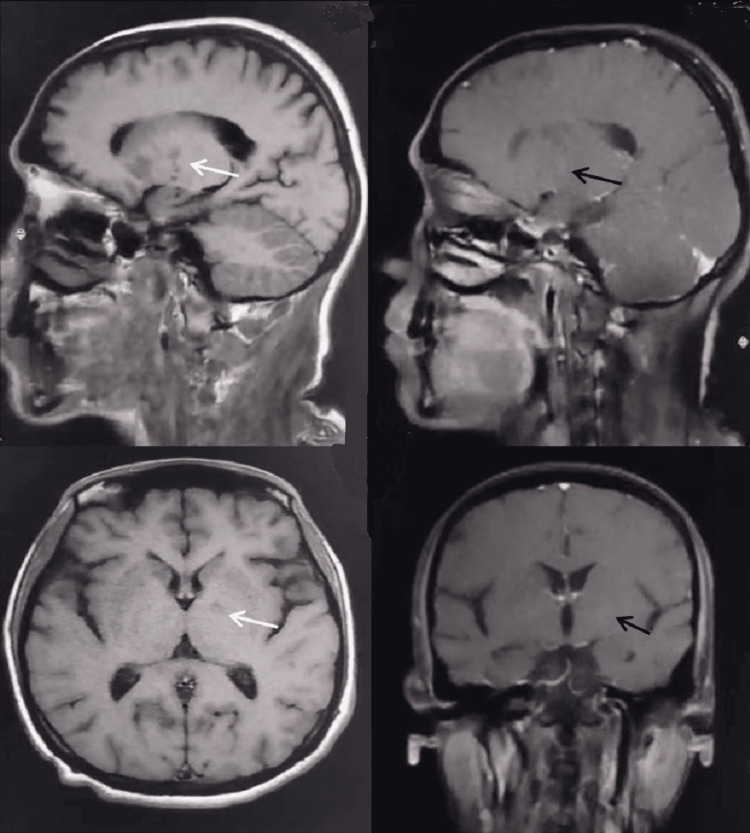
Pre-contrast T1-weighted imaging (white arrows) and post contrast T1-weighted imaging (black arrows) without evidence of enhancement

**Figure 5:**
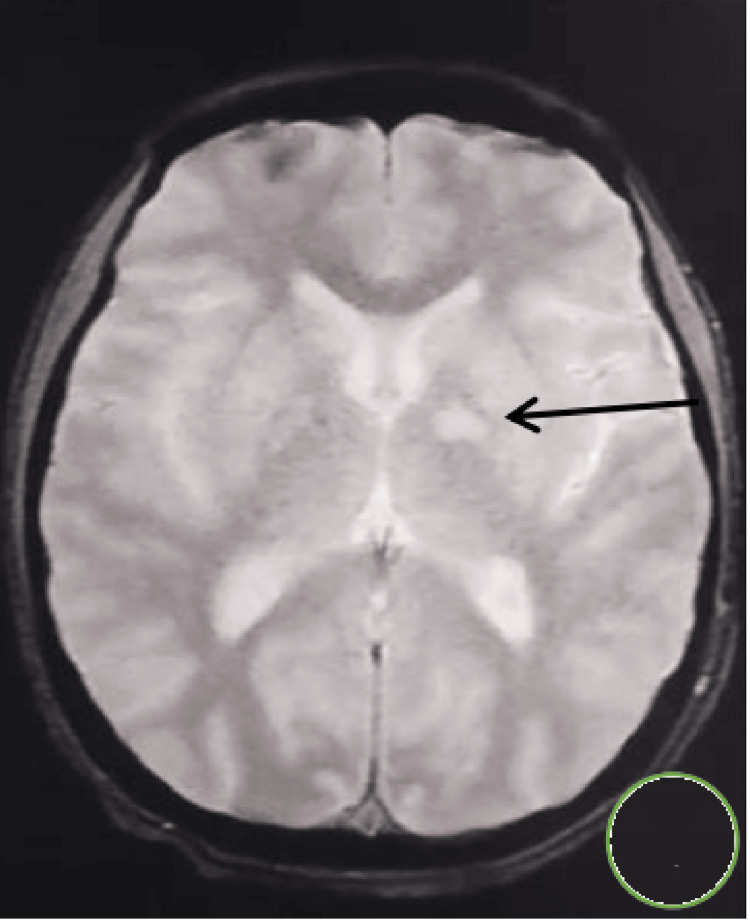
Susceptibility weighted imaging sequence at the level of basal ganglia shows no evidence of blooming artifacts to suggest secondary hemorrhage

Following confirmation of the diagnosis, the patient was referred to a specialized HIV treatment center for further management, and antiretroviral therapy (ART) was commenced in accordance with local treatment guidelines and protocol. Follow-up after referral to the HIV center was not performed, as the patient's condition was stable thereafter and did not meet criteria for admission.

During the admission course, the patient experienced three episodes of generalized tonic-clonic seizures within the first day, with complete recovery of consciousness after each episode. Additional seizure episodes occurred during the hospital stay but gradually declined in frequency. Seizure was controlled with levetiracetam and phenytoin. The total duration of hospitalization was two weeks.

## Discussion

Ischemic stroke is classified into five subtypes: large-artery atherosclerosis, cardioembolism, small vessel disease, stroke of other determined etiology, and stroke of undetermined etiology [8].

HIV is hypothesized to cause ischemic stroke at a younger age by multiple mechanisms, such as accelerated atherosclerosis, chronic inflammatory status due to HIV itself, or due to opportunistic infections. According to a cohort study done in 26,514 patients with HIV, 3.9% met the criteria for a potential stroke, with ischemic stroke comprising the majority of cases (75%) and small vessel ischemia representing 24% of ischemic stroke cases [9]. According to previous studies, small vessel ischemic stroke has been identified as the first or second cause of ischemic stroke in HIV patients.

Cerebral small vessel diseases (CSVD) are a group of syndromes related to the destruction of brain microcirculation, which comprises approximately 25% of ischemic stroke. Chronic infection with viruses such as SARS-CoV-2, HIV, hepatitis C, and Zika infections has been considered one of the contributors to CSVD development [10]. This case report describes CSVD as an initial clinical presentation for HIV infection, which is manifested as a white matter hyperintense signal on T2-weighted conventional brain MRI, which was observed in the left frontoparietal subcortex. White matter hyperintensities are considered a marker of CSVD severity in ischemic CSVD [11].

Individuals with HIV tend to develop stroke at a younger age compared to the general population, with a reported median age of 48.4 years in the US and even younger in lower-income countries (33.4 years in a study conducted in South Africa) [12]. This is consistent with our case, with an age of 35 years. Earlier onset of stroke in the low-income population may be due to delayed diagnosis and initiation of anti-retroviral therapy (ART) [12].

Presentation of stroke in the large majority of HIV-positive patients is not dissimilar to that in the non-HIV populations, but it tends to follow an atypical course over hours to days with variable presentation as altered mental status, fever, and confusion. In our case, fever and convulsion were the presenting symptoms.

In the context of convulsion and stroke, convulsion is a well-known complication of ischemic stroke and is classified as an early post-stroke seizure that occurs within seven days of ischemic stroke and a late seizure (according to the International League Against Epilepsy Definition). 

CSVD typically presents with convulsions, whereas most literature discusses other symptoms such as cognitive impairment and focal deficits. To our knowledge, convulsions have been described in CSVD patients over 60 years old with juxtacortical lesions.

In the context of initiation of ART, some studies have shown that it is associated with increased risk of developing stroke in the first six months, which may be due to an immune reconstitution inflammatory syndrome -like process, but decreasing incidence in the long term [13]. However, the patient was not on ART prior to presentation.

It is worth mentioning that opportunistic infections in HIV can also present with stroke-like symptoms. Stefaniac describes a case of a previously healthy patient who presents with left-sided hemiparesis, and the CT scan shows hypodensities involving the grey and white matter in the right basal ganglia, bilateral frontal lobes, and bilateral parietal lobes. In contrast to our case, multiple ring-enhancing lesions were detected in post-contrast CT, and a diagnosis of HIV with Nocardia abscess was made [14].

Literature also mentions HIV-associated cerebral vasculopathy that affects medium-sized blood vessels rather than small vessels and can manifest as cognitive impairment and encephalopathy [15]. 

This report has some limitations due to a single-patient description, which may limit its generalizability. Additionally, HIV follow-up tests, such as CD4 count, viral load, and cerebrospinal fluid (CSF) analysis, were not performed due to unavailability in low-resource settings.

## Conclusions

In this case, an undiagnosed HIV infection presented with multifocal lacunar infarctions and recurrent generalized seizures in a young adult without conventional vascular risk factors. Recurrent seizures, constitutional symptoms, and MRI evidence of cerebral small vessel disease led to the diagnosis of HIV infection. This presentation highlights the diverse neurological manifestations of HIV and the potential for HIV-associated cerebrovascular disease to be the initial clinical manifestation of infection.
